# To disclose or not: experiences of HIV infected pregnant women in disclosing their HIV status to their male sexual partners in Blantyre, Malawi

**DOI:** 10.1186/s12889-022-13974-4

**Published:** 2022-08-15

**Authors:** Annie Kalibwe Mkandawire, Vincent Jumbe, Alinane Linda Nyondo-Mipando

**Affiliations:** 1 Department of Health Systems and Policy, School of Public Health and Family Medicine, Kamuzu University of Health Sciences, Blantyre, Malawi; 2 Malawi College of Health Sciences, Blantyre, Malawi

**Keywords:** Disclosure, Male sexual partner, eMTCT, Malawi

## Abstract

**Background:**

HIV status disclosure is one of the pillars of success of the elimination of Mother to Child Transmission of HIV (eMTCT) program. However, there are challenges associated with it that limit full disclosure. Literature shows that for pregnant women in developing countries, who have been diagnosed with HIV, 16% to 86% disclose their status to their sexual partners. This study explored the experiences of newly diagnosed HIV-infected antenatal women in disclosing their HIV status to their male sexual partners in Blantyre, Malawi.

**Methods:**

This was a qualitative explanatory multiple case study that was conducted from 2018 to 2019 using in-depth interviews and diaries as data collection tools. We recruited seven newly diagnosed HIV pregnant women who had not disclosed their status to their male sexual partners and were initiated on Option B + strategy of the eMTCT of HIV at Limbe Health Centre. The investigator had 3 contacts with each participant from which data was gathered except for one participant who got lost to follow-up. This study employed content analysis and used a within-case and across-case analysis.

**Results:**

Women either use facilitated mutual disclosure process or disclosed directly to their male sexual partners. Women were motivated to disclose because they wanted an HIV-free baby, to know the partners' status, and to resolve the gap on how they got infected with HIV. The disclosure process faced challenges such as uncertainty about a partner’s reaction after disclosure, fear of relationship dissolution, and the soberness of the partner. Privacy was an important consideration during the process of disclosure. Following disclosure, male sexual partners either accepted the status immediately after disclosure or initially denied but later accepted.

**Conclusion:**

This study has shown that newly diagnosed HIV pregnant women accessing eMTCT services have a plan of either to disclose or conceal their HIV status from their male sexual partner and this decision is affected by the nature of relationship that exist between them and their partner. Factors relating to the unborn baby, the relationship as well as to know partners status motivate women to either disclose or conceal.

## Background

In 2014, a survey done in Malawi from 53 randomly selected immunisation sites estimated an HIV prevalence of 15.1% among postnatal women with newborns. Vertical transmission rate among them was at 8.5% [1]. Of the pregnant women admitted to antenatal ward at Queen Elizabeth Central Hospital (QECH), 30% of them were found HIV positive [2]. This shows the high burden of HIV among pregnant women, which can result into vertical transmission of HIV if eMTCT strategies are not adhered to [2]. In Malawi, new pediatric infections were at 3500 in 2018, a decline from 15,000 in 2010 which was attributed to the eMTCT strategy [2].

In 2011, Malawi introduced and implemented the Option B + strategy of eMTCT outside the WHO normative guidelines [3] . Evidence on the efficacy and cost-effectiveness of this strategy informed the WHO and led to the recommendation of the strategy in developing countries in the year 2013 [4].

Option B + is an approach that is currently being used in Malawi and other developing countries to prevent vertical transmission by initiating HIV-infected pregnant women on ART for life immediately after diagnosis, regardless of their CD4 count while their babies receive Nevirapine syrup for prophylaxis until they are no longer exposed to HIV [5]. Apart from the prevention of vertical transmission, this approach also offers advantages such as protection of sexual partner(s) because adherence to ART reduces viral load to undetectable levels where transmission is impossible [6]. In addition to that, ART also offers benefits to the woman’s health by boosting her immunity and preventing opportunistic infections hence leading to a healthy life for the mother [5].

Human Immunodeficiency Syndrome (HIV) status disclosure is the process of making one's status known to others, whether one is HIV positive or negative [7]. The global rates of HIV status disclosure vary according to locations with higher rates registered in developed countries compared to developing countries, ranging from 42 to 100%, and 16% to 86% respectively [8, 9]. The rates of HIV status disclosure among African women to their husbands range from 37% to 84.4% [10–13]. In Malawi, a study done in 2019 showed that disclosure rates by mothers enrolled in the elimination of Mother to Child Transmission (eMTCT) programme to their male sexual partners was at 94.5% [14].

Disclosure of HIV status benefits both the discloser and the disclosee [11]. Evidence suggests that disclosure reduces stress in individuals because it prompts the creation of a support system that optimizes access to HIV care, facilitates implementation of HIV risk reduction practices, assists in adherence to Antiretroviral drugs (ARVs), and improves uptake of eMTCT services [11, 15]. Evidence suggests that the reasons for disclosing an HIV status by women to their partners include resultant support including receipt of emotional, financial, or material support, preventing a partner from contracting the virus, enhancing trust in the relationship, and to promote the partners understanding of behavior change following an HIV infected diagnosis [16]. Notably, non-disclosure of an HIV-infected status to a male partner especially by mother accessing eMTCT services has several implications such as non-optimal ART treatment thereby increasing the rate of mother-to-child transmission of HIV [13–15]. Women fail to disclose their HIV-infected status because of perceived fear of stigma, blame, abuse, abandonment, violence and failure to find the need to do so [16].

Although HIV status disclosure is effective in improving the use of eMTCT services and promoting low rates of mother-to-child transmission of HIV (MTCT), women still experience challenges with the process of disclosure [12]. The challenges arise because of some past experiences of some women being stigmatised, abused, and having their marriages dissolved following disclosure of an HIV status [12]. Nonetheless, an earlier study showed that more women who were maintained in the Option B + program were the ones who had disclosed their HIV status to their male sexual partner because they were supported compared to those who were lost to follow up [17].

This study was guided by the Disclosure Process Model (DPM) which stipulates that disclosure of a potentially stigmatised identity such as HIV infection is dependent on the goal of disclosure or concealment [18]. The model asserts that for many, the disclosure event is not a one-time event but a continuous process. It further predicts conditions under which disclosure will yield a desirable reaction such as acceptance and social support from the disclosee or not. Finally, the model suggests that the outcomes of a single disclosure event affect the next disclosure process [18]. This model guided the study in the inquiry of why women disclosed their HIV status, how they disclosed, and the reactions which they received from their male sexual partners following disclosure. Given the relevance of disclosure to the effectiveness of the eMTCT program, this study sought to explore the experiences of newly diagnosed HIV infected women in disclosing their HIV status to their male sexual partner in the context of Option B + in Blantyre district, Malawi.

## Method

### Study design

This was a qualitative explanatory multiple case study that used in-depth interviews and diaries for data collection. This method was chosen because of its ability to explore in-depth an event or a process over a longer period considering that disclosure of an HIV status is a process, not a one-time event [17]. Furthermore, a case study design also permits a variety of methods to be used to understand a single issue hence in-depth interviews and diaries were employed to gather data of the women’s lived experiences in this study [19].

We employed in-depth interviews because of their ability to solicit required information such as feelings, beliefs, perceptions, and opinions which cannot be observed or written down. Additionally, because of the potentially stigmatizing nature of HIV, in-depth interviews offer a better way of gathering information as compared to soliciting information in a group environment [20]. We opted to include Diaries because they provided a better way of obtaining first-person observations of experiences that are recorded over some time [21]. In addition to that, participants were free to share what they wanted, as well as where and when without being burdened with recall since they recorded their experiences in real-time.

### Study setting

The study was conducted at Limbe health center in Blantyre, the southern part of Malawi. We selected Limbe health center because it is one of the health facilities in Blantyre, a district that has one of the highest HIV prevalence’s in Malawi (17.8%) [22]. At Limbe health centre, antenatal women are given HIV counselling and tested for HIV. Those who have tested positive for HIV are referred to ART clinic where eMTCT services are provided. The women are counselled on the importance of being enrolled in the eMTCT program. Following that, they are enrolled in the program, counselled on how to take ARVs, and are given a one-month supply of the drugs. For the first six months, the women are supposed to report to the health facility to refill the ARVs every month. This is done to ensure monitoring of the woman with drug compliance as well as side effects monthly for the first six months. After the first six months, when the ART providers are satisfied with the woman’s level of ARV compliance, the women are then given a two or three months’ supply of the ARVs and are requested to report to the health facility after every two or three months.

### Sampling and selection of study participants

We purposively selected seven women who were newly diagnosed with HIV, pregnant with an identifiable male partner whom they intended to disclose to or conceal from, and attending antenatal clinic (ANC) at Limbe health centre. We applied maximum variation when sampling by drawing women of varied gravidity. The women were interviewed when they came to access treatment after they had been attended to at the ANC and the ART clinic.

A total of seven women were enrolled into the study so that data was generated from each category of prim gravid, multigravid and multiparous women. However, one woman got lost to follow-up after the first interview but her available data was used during analysis. In qualitative studies, sample sizes are usually small, and case studies employ a minimum of 1 as a single case, and more than one as multiple case studies [23]. This is so because there are many bits of information in such studies and too many interviews would make analysis not to be thorough [23, 24]. Creswell suggested a sample of 5 to 15 in the case study research method hence this study employed seven participants so that data can be generated from each category of prim gravid, multigravid and multiparous women [23].

### Data collection

The interview guide was translated into Chichewa (Chichewa is a prominent language in the study area) for effective communication. The Principal investigator conducted all in-depth interviews and had 3 contacts with each participant from which data was gathered except for one participant who got lost to follow-up. Gathering information with participants in multiple contacts over a longer period helped the investigator to gain the trust of the participants cognizant of the sensitive nature of HIV infection, through a well-established rapport and hence gathering richer and accurate data [21].

The interview questions were developed and translated by the principle investigator and were reviewed by the second and third author. They interview guide was piloted before being used and the amends were made basing on the pilot so that they answered the research question. Questions on the first interview were slightly different from those of the second and third interview.

Participants were recruited at the ART clinic where privacy and confidentiality were easy to maintain. Firstly, the women were approached individually and when they gave consent, they were screened for eligibility which was followed by the initial interview. After the interview, participants were given a diary (or a tape recorder for those who did not know how to read and write) to take home where they recorded their experiences of the disclosure. The diary was in the form of a health passport book to preserve confidentiality. The women were also asked to bring the diaries during the second meeting which took place 4 weeks after the first meeting which was also their day of ART refill. This helped the investigator to capture the data and also to check if the documentation was taking place appropriately.

The second interview occurred after four to five weeks when women came for ARV refill. The interview focused on their disclosure experience including their feelings as well as the issues which they were not able to put down in writing in the diary. The inquiries also covered her partner’s immediate reaction after disclosure as well as his reaction days after disclosure and the challenges which they faced after the disclosure. We also asked for their suggestions to enhance status disclosure to partners in the eMTCT program.

The third interview occurred four to five weeks after the second interview; when each woman came to the facility for a refill of their ARVs. Participants were asked about how they felt having disclosed their status to partners, the challenges which they faced after disclosure, and the partner’s current reaction two months after disclosing. During this contact, the diaries were withdrawn from the women for data analysis. All interviews followed an interview guide and we probed for more information where necessary. A digital audio recorder was used to record all the interviews with the women.

This study ensured scientific rigor through triangulation which enhanced the credibility of the study [24]. This was achieved by the use of two different methods of data collection which are the use of diaries and in-depth interviews. This was done in order to gain a more and complete understanding of the women’s experiences. Additionally, the study employed respondent verification by checking with the participants if what was captured was really what they meant, by asking them to clarify what they meant during the previous interview that was came across when going through the transcripts. This was done to allow participants to verify their responses to solicit an accurate conclusion from the data collected [25].

### Data management

Audios were transferred onto a laptop that was password protected soon after each interview to ensure confidentiality. All the recorded data were transcribed verbatim by the principle investigator to prevent a change of meaning which was followed by a translation into English. The third author verified the transcripts against the audios to assess accuracy.

### Data analysis

This study employed content analysis and used a within-case and across-case analysis. This type of analysis makes a full and precise conclusion about a particular case or cases and seeks to describe a single item or case and to connect the unique aspects of a case with more general truths or principles [26, 27].

We deductively coded the data to identify quotations that were related to concepts in the conceptual framework (DPM) that guided the study [18]. We also inductively coded the data to identify codes that led to the formulation of new themes from the data that were outside of the model. The coding process was done at a phrase, sentence, and passage level. The related codes were recognized and new emerging themes were developed. This information comprises the steps that each participant went through during disclosure which included the time and how long after testing HIV positive when the disclosure event happened, how they disclosed, how they felt during the disclosure process, the challenges they faced during disclosure, the partners' reaction immediately, and days after disclosure.

The researcher summarised the key steps taken by the participant in their experiences of disclosing their status to their partner. Secondly, the researcher identified any patterns between participants. Specifically, the researcher looked for common behavior displayed by the women in their disclosure experiences, differences in the behavior, the context of the disclosure, common triggers for disclosure, content, and depth of information given to the partner during disclosure, successes and failures with disclosure, barriers/problems encountered during status disclosure and suggestions of what should be done to facilitate women’s disclosure to their male partner.

### Ethical considerations

Permission to conduct the study at the health center was sought from Blantyre District Health Office (DHO). Ethical approval to conduct the study was sought from the College of Medicine Research Ethics Committee (COMREC) (approval number P.02/19/2590). Written informed consent was sought from each participant before being interviewed. Identification of participants and all interviews were done in a private room at the health facility to ensure privacy and confidentiality. All data was anonymized by indicating numbers instead of names. All hardcopy data was kept in lockable cabinets and soft copy data in a password secured laptop to maintain confidentiality. Only those that were part of the study such as the investigator and the supervisors had access to the data. Since women were delayed in going home to participate in the interviews, refreshments were provided for them and they had their transportation costs reimbursed.

## Results

### Participant characteristics

The study involved 7 pregnant participants who were between the ages of 23 and 38 years. 6 participants were primary school dropouts while one was a secondary school dropout. The woman with the highest gravidity was 6 and the lowest was 1. The information has been summarised on Table 1.

**Table 1:** Demographic characteristics of participants

PARTICIPANT ID	GRAVIDITY OF PARTICIPANT	AGE	HIGHEST ACADEMIC LEVEL	OCCUPATION
1	4	30	Standard 4	Piece works
2	1	23	Standard 7	House wife
3	3	23	Standard 6	Small scale business
4	4	26	Form 2	House wife
5	4	38	Standard 5	Subsistence farmer
6	6	38	Standard 4	Local beer brewer
7	1	24	Form 2	Small scale business

#### Emerging themes

The main themes from the study were approaches for disclosure, environment for disclosure, factors that motivate disclosure, challenges with disclosure, and the reactions of the male sexual partner after status disclosure (Table 2).

**Table 2:** Key Themes and subthemes

Key Theme	Sub-Themes
Approaches for disclosure	Direct disclosure Facilitated mutual disclosure
Environment for disclosure	Suitable time and place for disclosure
Factors that motivate disclosure	Baby related factors: Potential for having an HIV uninfected baby and the wellbeing of the child Relationship Related Factors: a Loving Relationship, To know partners status, To protect the partner Quest for Freedom- Freeing oneself from fear of being caught with ARVs, Yearning for answers, To initiate mutual disclosure
Challenges with disclosure	Partner Related Factors: Fear of Relationship dissolution, Uncertainty about Partner Reaction,,The soberness of the partner
The reactions of male sexual partners after HIV status disclosure	Acceptance of the status and support of the partner Prompted partner to go for HIV testing- Accepts status of the partner but procrastinates HIV testing The initial denial of partner’s status

#### Approaches for disclosure

The findings showed that there were two disclosure approaches which were direct and indirect or facilitated mutual disclosure.

#### Direct disclosure

Some women decided to disclose their status directly to their partner through word of mouth which was either done through a face-to-face dialogue for those whose partners were around or telephonically for those whose partners were far away. One woman said the following when asked how she disclosed to her partner:

*“He greeted me and asked how my visit to the hospital was. So I told him that things did not go well, I have been found with HIV. He argued that how is that possible considering that I am negative. I said I do not know” (P5, interview 2)*

However, one woman did not feel comfortable to disclose telephonically because it was a borrowed handset and she decided to call the partner to come home for a face-to-face dialogue. She said the following:

*“I called him on the phone yeah! Telling him that you need to come quickly there is an issue here. So he asked that the issue which you can’t tell me on phone? I said that no I can’t explain the issue on the phone because this phone is borrowed” (P3, interview 2)*

Facilitated mutual disclosure.

Another method through which the women chose to disclose was facilitated mutual disclosure. One woman reported that she preferred this method of disclosure to ensure that the matter is taken seriously and avoid disbelief from the partner, because she could not communicate freely with the partner. She requested a health care worker to assist her with the disclosure process. The health worker called the woman’s partner and asked him to follow his wife to the health facility for HIV testing. Both the woman and the HIV counselor pretended not to know the woman’s HIV status. When asked why she called her partner to the health facility, one woman’s response was as follows:

“He should get tested, we should hear the results together. I will pretend as if I am being tested for the first time” (P2, interview 1)

On a follow-up interview, the same woman said:

“I called him to the hospital so he said he would come… When he came, we got tested again, we began the process once more” (P2, interview 2)

The woman further stated that she preferred this method of disclosure to allow the male sexual partner to be exposed to counseling at the hospital on HIV and care. It was also done to allow the partner to know their HIV status and be given the appropriate counselling and care depending on their status.

*“What happened, he was the one who received all the counselling and I was just listening. So I left everything in his hands. We went there, he received the counselling” (P2, interview 2)*

The woman stated that by having a test together, she would be assured that they will both start on treatment at the same time.

### Environment for disclosure

Participants in our study stated that there is a specific environment that is conducive to disclosure. Some women found the evening as a suitable time for disclosure because they usually discussed sensitive matters at that time after a husband has rested from work. Women believe that this would facilitate understanding of the issue being articulated and yield a positive reaction. One woman stated as follows:

"Yes! (Loudly) when he eats and he is full yes! …he should take a bath first, because he will be tired, he would be coming from work” (P6, interview 1)

Confidentiality was an important factor during disclosure. Women usually preferred to talk about their status while children and other members of the family were not around therefore the bedroom was a suitable place for keeping matters private. Additionally, one woman expressed concerns over children hearing their discussion about her HIV status and feared that the children may share the news with others which may result in unintended disclosure to others.

“Can you disclose on the sitting room where there are children? This needs to be discussed at a private place” (P6, interview 1)

However, the bedroom was the least preferred by some women in circumstances where privacy was compromised. One woman expressed concerns over the privacy of their bedroom as follows:

*“Because our rooms are like this (indicating the closeness of her bedroom room to that of her mother-in-law), side by side so when we are getting them (drugs) maybe when we are discussing she hears us” (P2. Interview 3)*

### Factors that motivate hiv status disclosure or concealment

The factors that motivate women to disclose their HIV positive status to their male sexual partners were baby related factors which included potential for having an HIV uninfected baby, wanting to know the partners’ status, the need to protect the partner; Quest for Freedom which included freeing oneself from fear of being caught with ARVs, yearning for answers, initiation of mutual disclosure. Relationships that were deemed insecure motivated concealments of an HIV status.

### Factors relating to the baby

#### “We can protect our child”-Potential for having an HIV uninfected baby

Women were motivated to disclose their HIV status because of the desire for a healthy and HIV-free baby. Women expressed a desire to be supported by the husband in different ways to prevent transmission of the virus to their child, hence they opted to disclose their status to gain that support. Some women stated as follows:

“So that he can start taking the drugs soon so we can protect our child” (p3, interview 1)

“The most benefit is for the baby that I am expecting, he/she is the one that will benefit more. (p2, interview 1)

#### “The child needs to be taking drugs”-The well-being of a child

Women often weighed the importance of disclosing the status to their partners over the challenges associated with concealing their status especially when their child is born considering that the child will also require prophylactic drugs such as Nevirapine. As such, the administration of prophylaxis to a child enhanced their decision to disclose their status to their male sexual partner. One woman said the following:

*“What prompted me was that I sat down and thought about it that even if I hide it there is nothing that can ever happen to the way I am. It will happen that the child is born and needs to be taking drugs so I need to tell him so that he knows the truth, if he too is like that we should be moving together, if the other one is okay then we shall see what shall happen. That’s why I gathered the courage” (P4, interview 2)*

### Factors related to their relationship

#### “He loves me”- A loving relationship

One women pondered over the goodness and loving nature of the husband and concluded that even if she disclosed her status to the husband, he would easily accept her status and hence he will not divorce her. She had the following to say:

*“Looking at the way we lived [atmosphere within the home], I saw that he loves me, so even if I tell him I thought that maybe he can’t change his thoughts, or maybe becoming furious”(P4, interview 2)*

#### “Truth sets you free”- Freeing oneself from fear of being caught with ARVs

Women reported being uncomfortable with concealing their HIV positive identity from their partner and preferred to make their husband aware of their status. They stated that this will enable them to take their drugs openly and in liberty without being cautious of being caught with the drugs in the house. Some women had the following to say:

“The reason was that he should know. For me to be doing everything in secret I saw that it would not be good” (P5, interview 2)

“That is why they say that the truth sets free, so it is better that you should stand on the truth it is up to him to accept it or not” (P4, interview 1)

#### “Maybe he might stop visiting me” insecure relationship

One woman planned to conceal her status to her sexual partner because she thought that disclosing will affect their relationship which was already in a mess due to other issues. She had the following to say:

*“in the past he used to come each and every day, but when he just knew that I am pregnant and the pregnancy is growing he stopped coming frequently, he takes sometimes 3 days without visiting me… if I disclose maybe he might literally stop coming”(P1, interview 1)*

### Factors relating to wishing to know partners status

#### To know a partners HIV status

Women said that they opted to disclose their HIV status so that they also get their partners to test and know their status with a three-fold aim of protecting the partner if he is uninfected, to know the source of the infection, and to facilitate mutual disclosure. One woman had the following to say:

*“And also my husband, maybe he might be the one who has infected me and he was hiding it from me, maybe he takes the drugs, so maybe he fails to tell me, he was hiding it. That’s the reason why I would like to disclose to him so that if he is like that he should be free to tell me” (P4, interview 1)*

#### “Take care of him”- To protect the partner

Women disclosed their HIV-positive status because they wanted their male sexual partner to go for an HIV test so that if they are found negative they can be able to protect themselves from getting infected.

*“…it is better to tell him so that he can go for testing earlier so that if he does not have the virus he should be able to take care of himself, or if he is found with it he should also take care of himself” (P3, interview 3)*

Furthermore, women wanted to ensure that the partner starts their treatment earlier if they are found with the infection.

*“So I saw it very wise to just call him we should get tested together we should both know if we are the same or if we are different. So that we both start taking the drugs the same day” (P2, interview 3)*

#### “I was okay!”- Yearning for answers

Women disclosed as a way of getting disclosure on how they got infected because some wondered how they got infected. Some women claimed that they were HIV negative before getting married to their partner and thought that it was the husband who infected them. One woman said the following:

*“How is it possible for me to be positive? Because in the past, when I was single I used to go to the hospital to get tested, even the counselors who were moving in the areas these years, tested me and I was ok. Sure. So I started antenatal clinics and I was found to be this way, that kept me wondering and worried, even the time I was talking to you, I had so many questions I was just being strong” (P2, interview 3)*

One woman was convinced that she was infected by the husband and that gave her the confidence to disclose her status.

“No I do not have any fears, I thought he is the one who has given me the disease, am I promiscuous?” (P6, interview 1)

#### “He was hiding it”- To initiate mutual disclosure

Women also thought that disclosing their HIV positive status to their partner was a better way of facilitating mutual disclosure within a couple. This would lead the husband to disclose his status if they are infected. Some women were convinced that they had been infected by the husband who was unable to inform them of his status. This conviction encouraged them to disclose.

*“I had already tested and my blood was ok, I had no problem. As I was meeting this man I had no problem. But now I am being found with the problem. Meaning that he is the one who has infected me. This is the reason why I am courageous enough that I will disclose to him… he might be the one who has infected me and he was hiding it from me, maybe he takes the drugs, so maybe he fails to tell me, he was hiding it. That’s the reason why I would like to disclose to him so that if he is like that he should be free to tell me” (P4, interview 1)*

### Challenges with disclosure

The challenges that women faced with disclosure were uncertainty about partner reaction and dilemmas of whether to disclose or not because of fear of being divorced.

#### “He might say you infected me”- Uncertainty about partners reaction

Women stated that their uncertainty about a partner’s reaction following disclosure often resulted in deciding against disclosure of an HIV positive status to a partner. One woman said the following:

*“So I just think that maybe if I disclose he might say that you have infected me, while maybe he is the one who has infected me. Maybe he might stop visiting me because even if he comes I do not depend on him. Now I depend on myself, I find some piece works to find food. That is why I doubt that I will tell him, maybe I should just be knowing myself” (P1, interview 1)*

#### “he would divorce me”- Fear of dissolution of a relationship

Some women reported having refrained or hesitated on disclosing because they feared desertion by the partner once he learns that she is HIV positive. Two women wrote in their diaries:

“I thought that he would divorce me when I disclose” (P7, diary)

“The day I disclosed my status to my loved one I was so afraid, I thought that he might divorce me” (P6, Diary)

To concur with her, another woman thought when the sexual partner learns of her HIV-positive status he would terminate their relationship. This came about because he was already thinking of leaving her because of the unexpected and unplanned pregnancy. The woman said the following:

*“Because in the past he used to visit me every day, but currently, when he saw that the pregnancy is growing he does not come often. Sometimes 3 days pass by without him coming… so maybe if I disclose he might permanently stop coming” (P1, interview 1)*

#### “he would say disappointing and rude words”-Fear of disappointing reaction

One woman feared that the partner would say things that are rude and disappointing after learning of her HIV positive status. She narrated her fears in the diary and interview as follows:

“I was afraid to tell him at first” (P3 diary) “I thought he would say things that are disappointing and rude” (P2, interview 2)

#### “This is not the story to discuss while someone is drunk” – The soberness of the partner

One woman reported that she was not comfortable to disclose her status to her husband whilst he was drunk but preferred to wait until the man was sober to ensure that he reacts to the news whilst he was in his right frame of mind. She thought that disclosing her status to her husband whilst he was drunk would irritate the husband and make him overreact over the issue and consequently this delayed disclosure in one woman.

*“I can say that it took 3 days huh? Yes. All those days he was coming home drunk so I figured that eeeh!, this is not a story to discuss while someone is drunk, it can reach to an extent of fighting… So the day that he was ok that is when I told him that” (P6, interview 2)*

### The reactions of male sexual partners after hiv status disclosure

Six women reported that their partners accepted the HIV positive test results and took varying actions afterward. Following acceptance, women reported that their partners offered support to the woman, took an HIV test whilst others procrastinated on having an HIV test. One woman stated that her partner denied the results initially but later accepted them.

#### “He reminds me”- Acceptance of the status and support towards the partner

After receiving the news about the partner HIV positive status, men encouraged their partners in different ways. Sexual partners supported their partners with drug compliance by reminding them to take drugs at the required time. Most men reminded their partners to take the ARVs when they haven’t taken them or give the drugs to their partners so that they can take them. In addition to that, they also gave them words of encouragement as regards to drug compliance.

“He reminds me every night. When I just take supper he reminds me to go and take the drugs so I take them and drink them” (P4, interview 3)

*“But I thank God because I and my husband we stay happily. There is no problem between us and he encourages me to take my drugs because we are expecting a healthy child” (P6, diary)*

#### “He went for testing”- Prompted partner to go for HIV testing

Following disclosure, some women reported that other men decided to go for an HIV test so that they may know their status as well. Out of the six men whose partners were involved in the study, four went for HIV testing in response to receiving news about their wife’s status. Of those who went for testing, three were found HIV negative while one was found HIV positive. When asked how her male sexual partner responded following her HIV status disclosure, one of the women said:

“He accepted it, but he responded the following day and he accepted that he was going to get tested so he went for testing” (P7, interview 3)

“When I disclosed he accepted it and went for testing but he was found to be negative” (P6, diary)

#### “But days are going”- Accepts status of the partner but procrastinates HIV testing

Two women reported that their partners had accepted the HIV status of the partner yet they procrastinated their testing upon being advised by their partner. They responded that they had accepted the status of the wife, encouraged them to comply with the treatment, and promised to go to the health facility for an HIV test but never went for testing until the end of the study.

“When I tell him to come for testing he just says he will come so I do not know when he shall come, but days are going” (P3, interview 3)

#### “He argued”- Initial denial of a partner’s status

One woman reported that after disclosing her HIV positive status, her partner was in a state of unbelief at first. He wondered how his wife got infected when he tested negative the previous month. He was worried about the outcome of the child since it was at risk of contracting HIV. However, he eventually accepted the status of the partner and encouraged her to comply with treatment so that the child should not contract HIV. He got another HIV test and was also found HIV negative.

*“he said that it is not possible because last month I was there and I tested HIV negative…. he said aaa it could have been better if you were ok, but there is a risk to the child. ” (P5, interview 2)*

## Discussion

This study explored the experiences of newly diagnosed HIV positive antenatal women in disclosing their HIV status to their male sexual partners in Blantyre, Malawi. The main findings of the study were that women disclosed directly or through facilitated mutual disclosure. The bedroom was the preferred place for disclosing the results. The motivating factors for disclosing an HIV positive status to male sexual partners were potential for having an HIV uninfected baby, the wellbeing of the child, having a loving relationship, wanting to know the status and protect the partner, freeing oneself from fear of being caught with ARVs, yearning for answers, and as a measure of initiating mutual disclosure. The challenges associated with disclosure included uncertainty about partner reaction, fear of relationship dissolution, and the soberness of the partner. The reactions of men following disclosure were acceptance of the status and offering support while others denied the status and further procrastinated their testing.

In our study, women explicitly stated their status to their husband to avoid uncertainties and to gain the support which they needed. Our finding on direct disclosure of HIV status through word of mouth differs with results from a study done by Bhatia where women disclosed through word-of-mouth but used words that did not explicitly state that they are HIV positive [28]. This kind of disclosure however, may be risky as it may bring about intimate partner violence during the time of disclosure. Previous studies report that couple testing is an evidence-based strategy that has proved to yield successful disclosure in couples, and women use it to indirectly disclose to their male sexual partner [28, 29].

Our study reported that one woman asked a health worker to facilitate a couple testing through a phone call to the partner, who later reported to the facility. This method is known for its effectiveness in persuading a male sexual partner to come for testing and it improves rates of status disclosure [30, 31]. The use of phone calls has yielded positive outcomes in HIV service delivery. Previous studies have shown it to bring a 40% increase of repeat HIV test following reminders through texts messages and phone calls, and an increase in drug adherence rate from 87% to 94% among people living with HIV [30, 32]. As such, eMTCT health providers may use this method to invite a male partner for a session that will also include facilitated mutual disclosure of HIV status among couples. This method has to be exercised with caution as it may apply only to those whose partners have a mobile phone. Additionally, Bhatia et al argued that issues of gender-based violence should also be considered before health workers involvement in phone calls for couple testing [28]. According to a study done by Walcott et al in Kenya, facilitated disclosure has several advantages which include improved ability to accept HIV results by the sexual partner, enhances understanding of HIV and treatment, improves drug adherence, and reducing HIV related fears because of the counseling and the information that is given during the process [33].

Privacy and confidentiality were important factors when it came to the place and the time for the disclosure of their HIV status in our study. According to our study, these factors determined whether disclosure was done immediately after learning HIV positive status or was delayed. It also determined where disclosure took place and the bedroom was preferred for most women in our study. This was done to prevent those who women did not intend to disclose to such as child, mother in-law and sister from hearing the news. While there ample literature showing the need for health workers to disclose to clients their status in private, there is scanty literature suggesting privacy and confidentiality as a requirement during disclosure of HIV positive status by eMTCT women to a male sexual partner [34].

Existing literature shows that HIV disclosure is facilitated by factors such as knowledge of partner’s HIV status, the number of sexual partners, and relationship status [35]. Our study however found different facilitators to HIV status disclosure. Firstly, women considered the wellbeing of the baby which was earlier asserted by Bhatia et al. who reported that although disclosure motives differ by gender, women often consider the benefits of disclosure and mostly disclosed to gain support from the partner in an attempt to prevent perinatal infection [28]. In a different study in Ethiopia, Lifson found a positive association between disclosure and social support which included guidance/advice and material aid [36].

As was reported in an earlier study [37], our study also found that women disclosed to their partners so that they are supported. Notably, similar to our study and an earlier study also reported that women refrained from disclosing because they wanted to maintain the relationship with the partner in fear that the relationship might end as well as fear of stigma [37]. Although women in our study had this trigger to disclose, they were also afraid of the implications of disclosure to their partner as they were afraid of divorce and accusations of infidelity. This finding remains consistent with results from a study that was done by Adam et al in Ontario. In this study, it was found out that although participants indicated that disclosure had its benefits of gaining support from a partner, it is also a means of setting oneself up for rejection and stigmatization by the partner and the community [38].

This kind of fear is one of the reasons why only three women in our study disclosed on the same day of their HIV diagnosis while the other women took three to ten days to disclose. In a different study by Sendo, 65.4% of women disclosed to their sexual partner in less than one month after being diagnosed HIV infected, while others took up to six months to disclose [39]. Women delay disclosure as they thought that disclosure should be done gradually [31]. Additionally women also delay disclosure in fear of loss of social support, economic security and stigma [28]. However, women in our study finally disclosed as they saw that the benefits of disclosure outweigh the fear which they had about the negative consequences of HIV disclosure. A study by Watt reveals that in an attempt to avoid stress, some women chose not to disclose their status until the baby was born as a measure of averting stress to the sexual partner [40]. These differences in factors that precipitate disclosure events may arise due to differences in disclosure goals as stipulated by the disclosure process model by Chaudoira [18].

The atmosphere of the relationship prompted women in our study to disclose their HIV positive status to their partner which is consistent with a study by Hino et al, who reported that women felt the urge to disclose their HIV positive status to their partner because of the love and commitment which existed between them and their sexual partner [41]. However, in the same study, fear of stigma prompted some not to disclose [41]. According to other studies, a decision of whether to disclose HIV positive status to a sexual partner or not is largely facilitated by prior knowledge of the partner's HIV status and the quality of the relationship that exists between the woman and the partner, and whether married or not [35, 42]. In our study, knowledge of partners' HIV status was not explored from the participants before they disclosed their HIV positive status to their male sexual partner.

Our finding that women opted to disclose their HIV status so that they can take their ARVS overtly builds on what was reported in a study by Naigino where it showed that women who conceal their status were covertly taking their ARVS and also failed to comply with their hospital appointments [13]. This results in ART noncompliance which in turn leads to an unsuccessful eMTCT program [13].

The finding that women disclosed their status to trigger mutual disclosure in order to learn their partner’s results is in contrast to findings from Canada where they found out that gay men thought that it was a partner’s responsibility to find out the partner’s status and protect themselves from being infected with HIV [38]. While results from Canada correlate with the views of one participant of our study, who was in a casual sexual relationship with the partner, these views are different from the views of the other six participants of our study who were in a marriage relationship. The type of relationship with the partner therefore might determine disclosure or non-disclosure of HIV status [42, 43].

The main challenges with status disclosure were uncertainty about partner reaction and fear of marriage dissolution. This finding supports assertions by other researchers who found out that disclosure is more likely to occur in more established relationships as compared to unestablished ones [44]. The fear of marital dissolution as stated in this study is in line with the results from a previous study that reported that fear of stigma and relationship dissolution also influenced non-disclosure among couples [28]. Notably, women often associate disclosure of their HIV infected status with the dissolution of their relationship [31, 45]. This may explain why one woman in our study reported that she planned to hide her HIV status and her ARV from the partner as she thought that disclosing would lead to the automatic dissolution of her relationship and accusations of promiscuity. Literature has shown that this kind of behavior often leads to ART non-adherence as a result of underutilization of eMTCT services through drug noncompliance and missing appointments in an attempt to conceal HIV status [46]. For this specific woman in our study, her insecurities stemmed from the poor relationship which existed between the two. Her relationship with her sexual partner was not an established relationship in comparison with other participants in the study who disclosed their HIV status. This finding builds on previous assertions that state that disclosure is more likely to happen in more established relationships as compared to unestablished ones [44].

In a study by William, intimate partner violence is exacerbated by alcohol intoxication. Women who experience intimate partner violence are filled with certainty about partner behavior when drunk [47]. This might be the reason why one woman in our study delayed HIV positive status disclosure until partner was sober in order to avert violence by the partner. Notably, a previous study reported that some participants regarded non-disclosure of HIV-positive status as the best way of overcoming stigma and marriage dissolution [28]. However, for most women in our study, disclosure was the only way to overcome their fears, to gain personal freedom as well as support from their partner. Additionally, it has been asserted that those people who disclosed their HIV positive status often received a positive response from their sexual partner [29, 36]. The partners would offer encouragement to adhere to ART and provide transport to ensure that she does not miss her hospital appointment dates [29]. Additionally, the disclosure process model by Chaudoir also states that disclosure of identity often yields positive results than concealment [18]. In contrast to this, the study by Van Lettow in Malawi shows active discouragement to ART drugs by the partners because of the side effects that come along with them [14].

Male sexual partners in our study either accepted the status and went for testing, denied and never got tested, or delayed the HIV test. When men fail to go for HIV tests, it means that they fall short in male involvement and male involvement is a vital component in increasing the success of the eMTCT program [48, 49].

We noted that good planning of the disclosure event by women in the eMTCT program is a factor that may have resulted in positive disclosure outcomes in our study. Women who had plans to disclose planned how they would go about it beforehand. They thought of the right time and place for the disclosure and they carefully chose words they would use during HIV status disclosure [18]. Inability to plan disclosure may prevent women from disclosing and having a positive response from the partner after disclosure [18]. It is therefore important that women be enlightened and guided on how best they can break the news of their HIV infected status to their partner [18].

Our study did not report any forms of partner violence after disclosure but another study that was done in Malawi, South Africa, and Tanzania reported intimate partner violence in forms of physical abuse, being denied financial support, and/or marriage dissolution [50]. The absence of any forms of partner violence in our study may be attributed to an increase in knowledge about HIV issues amongst people. Another factor may be the small sample size which limited the number of disclosure events hence limiting the number of disclosure outcomes. Lastly, it might be the short time frame within which the research was done of which partner response towards the HIV status of the partner might change over time.

### Strengths and limitations of the study

Interviewing participants three times at different times ensured that trust was gained from the participants which ensured the opening up of the women as regards the stigmatizing nature of HIV hence obtaining rich data. This was done by doing the study over a longer period and having multiple contacts with each woman. The study had several limitations. The first was our failure to track a participant who had been lost to follow-up after the first interview which made it impossible to access her additional data. To resolve this, her available data was still used for analysis and also an additional participant was enrolled making the total sample size of 7. Secondly, some participants would miss their appointment dates and come on a day when the interviewer was not available. The HTC providers were helpful here as they would inform the investigator when the participants come. Other participants would forget their diaries at home when coming for their appointments; this made it difficult for the interviewer to capture data on time. Another challenge faced was that the participants did not use the diaries as required. Some of them only used them once, after disclosure. The last challenge was that the point of enrollment of participants into the study did not allow non-disclosure to be known before-hand hence there was no control of number of non-disclosure experiences to be included in the study.

## Conclusion and recommendations

This study has shown that newly diagnosed HIV pregnant women accessing eMTCT services have a plan of either to disclose or conceal their HIV status from their male sexual partner and this decision is affected by the nature of relationship that exist between them and their partner. Factors relating to the unborn baby, the relationship as well as to know partners status motivate women to either disclose or conceal.

Health workers in low and middle income countries need to know the motivation and discouragement factors of disclosure. This will enable them to counsel women so that they can disclose, and how best they can do it in order to receive a positive feedback from their male sexual partner which will lead to male support, an important component in the uptake of PMTCT services. Therefore, health systems need to have multiple pathways that can be offered to a woman to assist them in the disclosure process.

Policymakers should consider making policies that would strengthen facilitated mutual disclosure where men can be asked by the health workers to come for HIV testing and counseling with their wife. Health providers may consider assisting or counseling women on how best to disclose to their male sexual partner. Further research studies are needed to find out long-term outcomes of relationships between women living with HIV and their HIV uninfected male sexual partner and also how this affects their future reproductive goals.

## Data Availability

The datasets used and/or analysed during the current study are available from the corresponding author on reasonable request.

## Abbreviations

AIDS: Acquired immunodeficiency syndrome
ANC: Antenatal clinic
ART: Antiretroviral therapy
ARVs: Antiretrovirals
COMREC: College of medicine research ethics committee
DPM: Disclosure process model
HIV: Human immunodeficiency virus
MTCT: Mother to child transmission
eMTCT: Elimination of mother to child transmission
